# Effects of corneal crosslinking on corneal shape stabilization after orthokeratology

**DOI:** 10.1038/s41598-020-59157-2

**Published:** 2020-02-11

**Authors:** Chimei Liao, Xingyan Lin, Stuart Keel, Jason Ha, Xiao Yang, Mingguang He

**Affiliations:** 1 State Key Laboratory of Ophthalmology, Zhongshan Ophthalmic Center, Sun Yat-sen University, Guangzhou, China; 2 Center for Eye Research Australia; Ophthalmology, Department of Surgery, University of Melbourne, Melbourne, Australia; 3 Faculty of Medicine, Nursing and Health Sciences, Monash University, Clayton, Australia

**Keywords:** Refractive errors, Translational research

## Abstract

Orthokeratology (Ortho-K) works to reshape cornea and is the only non-surgical way to enable vision without corrective aids. However, its effect is only temporary, and successful stabilization requires ongoing Ortho-K wear to maintain the reshaping effect. Corneal crosslinking (CXL) is a commonly-used technique in clinical practice to stabilize corneal shape in keratoconic eyes. However, whether or not CXL can stabilize corneal shape after Ortho-K in normal cornea has not been reported. Therefore, this proof-of-concept study using 2 rhesus monkeys aimed to determine the efficacy of the combined procedure. One monkey wore Ortho-K bilaterally for 24 hours, and the other from 6 pm to 8 am for 7 days. The left eyes of both monkeys underwent CXL after Ortho-K while the contralateral eye served as control. Results showed a gradual regression of corneal shape in all eyes with or without CXL. However, eyes underwent CXL regressed more slowly than the control eyes. The control eyes and the CXL treatment eye in the 7-day Ortho-K monkey regressed completely at last, while the CXL treatment eye in the 24 h Ortho-K monkey maintained a corneal flattening of −1.48 D 27 days after procedure. These findings suggest CXL can slow the regression of Ortho-K for a short duration, but cannot sustain its effect according to the current protocol.

## Introduction

As a non-surgical vision correction and with the potential to reduce myopia progression^1^, Orthokeratology (Ortho-K) has received much attention in the past decades. Correction of myopia is achieved as Ortho-K thins and functionally flattens the central cornea of the wearer during sleeping. However, the effect is only temporary, thus successful stabilization requires ongoing Ortho-K wear to maintain the reshaping effect, which comes at an inconvenience and cost for users.

Corneal crosslinking (CXL) is a clinical procedure designed to slow down or halt the progression of keratoconus. While the precise mechanism is yet to be elucidated, the molecular mechanisms accounting for the corneal change may be attributable to the cross-linking of collagen fibrils through the photochemical reaction between ultraviolet A (UVA) light and photosensitizers^2^. Previous studies have shown a significant increase of up to 70–300% in corneal rigidity after CXL^3,4^, suggesting the potential of CXL to stabilize corneal shape. Though originally proposed as a treatment for keratoconus, the use of CXL has been recently extended in LASIK surgery. With proven evidence that CXL is able to achieve improved stabilization of corneal refraction after refractive surgeries^5,6^, its potential as a promising auxiliary treatment to stiffen corneal shape has invited much attention.

Ortho-K is able to reshape the cornea to successfully correct myopia, thus whether or not CXL is able to sustain its reshaping effect and maintain the correction of refractive errors in patients after Ortho-K is of substantial clinical value to investigate into. There have been efforts to apply this combined therapy in keratoconus patients^7,8^. Results showed that the visual acuity of keratoconus patients improved after this combined therapy, but no effects of corneal stabilization were observed. However, the abnormality of collagen structure and loss of collagen fibrils in keratoconus may have weakened the efficacy of both Ortho-K and CXL^9^. As the effect of this combined procedure remains unknown in normal corneas, in this proof-of-concept study we aimed to investigate whether CXL can stabilize corneal shape molded by Ortho-K lenses in normal cornea using rhesus monkeys.

## Results

### Slit lamp microscopy

Given the fact that the shapes of both eyes in our monkeys were not identical, optimal lenses were selected for each eye with only slight differences in fitting curve parameters. The Ortho-K lenses were well-fitted and well-tolerated by both monkeys. No epithelial fluorescein staining was observed after the removal of Ortho-K lenses in either eye of both monkeys. Corneal epithelial defects in the CXL treatment eyes were healed by the fifth day post CXL procedure. Mild to moderate corneal edema or stromal haze gradually improved after corneal epithelial healing and completely recovered 10 days following the procedure.

### Corneal topography and refractive power change

**Figure 1:**
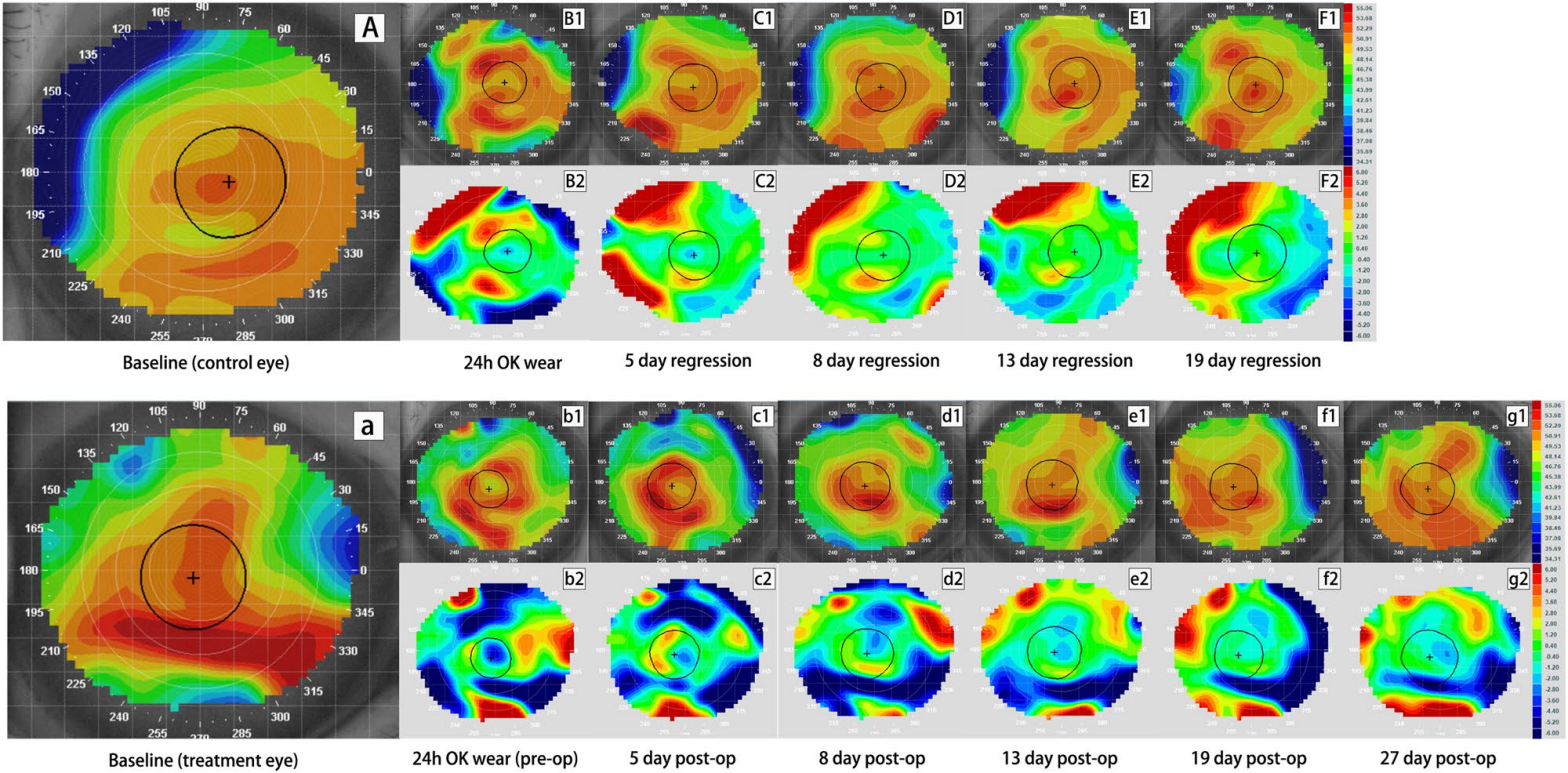
Tangential and subtractive maps of the control and crosslinking (CXL) eye from monkey A wearing Ortho-K lens for 24 h. Picture A–F_2_ are profiles for the control eye while picture a-g_2_ are profiles for the CXL eye. Picture (A),(a) are the baseline tangential maps of the control and treatment eye, respectively. Picture B_1_ and b_1_ are tangential maps after wearing Ortho-K lens for 24 h. Picture C_1_–F_1_ show corneal shape at 5 days, 8 days, 13 days and 19 days after lens removal while c_1_-g_1_ show corneal shape at 5 days, 8 days, 13 days, 19 days and 27 days after CXL. Picture B_2_–F_2_ and b_2_–g_2_ are corresponding tangential subtractive maps compared to their baseline (B_2_ was generated from B_1_ minus A and b_2_ was generated from b_1_ minus a, etc.), representing curvature change at each visit relative to original shape. Warm colors (red) indicate corneal steepening while cold colors (blue) indicate flattening in subtractive maps. Central corneal flattening was achieved in both eyes after wearing Ortho-K lenses (B_2_ and b_2_), followed by gradual regression (C_2_–F_2_ and c_2_–g_2_). The control eye regressed completely by 8 days (D_2_), while the CXL eye maintained a −1.48 D of corneal flattening at 27 days post-operatively (g_2_).

**Figure 2:**
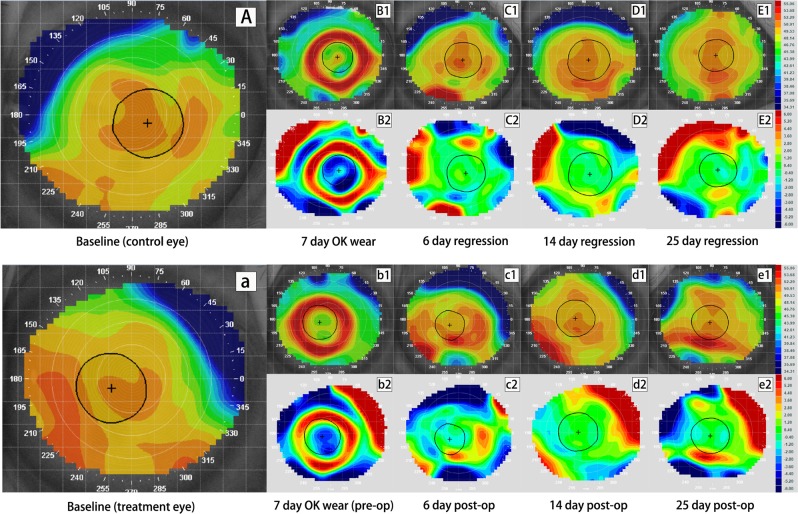
Tangential and subtractive maps of the control and crosslinking (CXL) eye from monkey B wearing Ortho-K lens for 7 day. Picture A–E_2_ are profiles for the control eye while picture a–e_2_ are profiles for the CXL eye. Picture (A),(a) are the baseline tangential maps of the control and treatment eye, respectively. Picture B_1_ and b_1_ are tangential maps after wearing Ortho-K lens for 7 days. Picture C_1_–E_1_ show corneal shape at 6 days, 14 days and 25 days after lens removal while c_1_–e_1_ show corneal shape at 6 days, 14 days and 25 days after CXL. Picture B_2_–E_2_ and b_2_–e_2_ are corresponding tangential subtractive maps compared to their baseline (B_2_ was generated from B_1_ minus A and b_2_ was generated from b_1_ minus a, etc.), representing curvature change at each visit relative to original shape. Warm colors (red) indicate corneal steepening while cold colors (blue) indicate flattening in subtractive maps. Central corneal flattening was achieved in both eyes after wearing Ortho-K lenses (B_2_ and b_2_), followed by gradual regression (C_2_–E_2_ and c_2_–e_2_). The control eye regressed completely by 6 days (C_2_), while the CXL eye regressed completely by 14 days post-operatively (d_2_).

**Figure 3:**
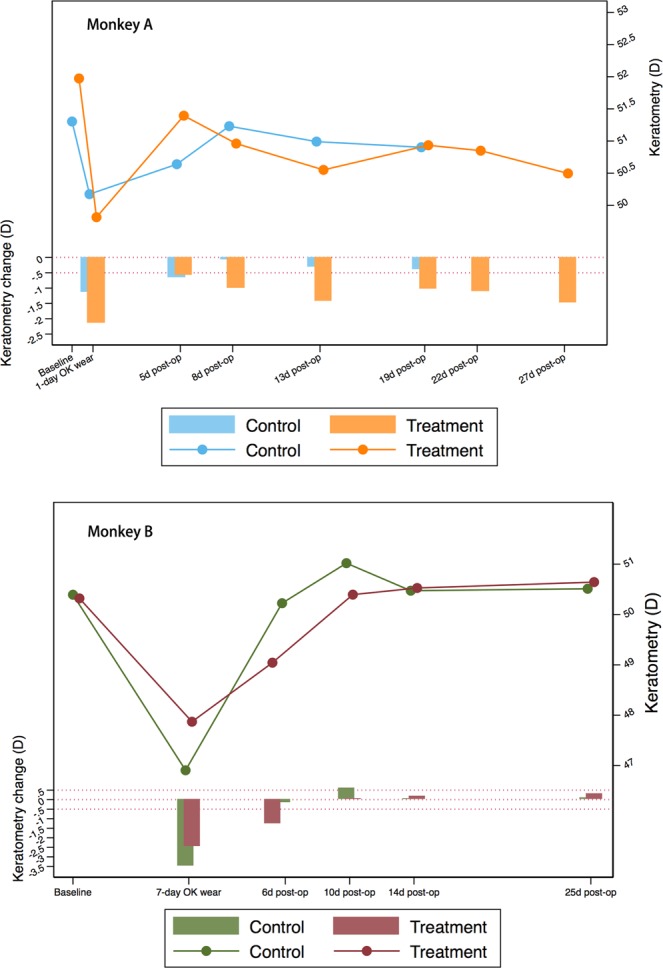
Mean keratometry changes of treatment zone in monkey A wearing Ortho-K lens for 24 h (top) and monkey B wearing Ortho-K lens for 7 days (bottom). The line chart shows the change of mean keratometry values (D) at each visit while the bar charts presents the difference of keratometry (D) at each visit compared to the baseline level. Reference line: y = 0 and y = −0.5. For monkey A, keratometry change in the control eye was less than −0.5 D at 8 days after lens removal, while the CXL eye maintained a −1.48 D of corneal flattening at 27 days post-operatively. For monkey B, keratometry change in the control eye was less than −0.5 D at 6 days after lens removal, while the CXL eye regressed completely at 10 days after post-operatively.

The corneal tangential maps and corresponding subtractive maps of the 2 monkeys are presented in Figs. 1 and 2. Their corresponding value of change in mean keratometry within the reshape zone was shown in Fig. 3. In Monkey A that wore Ortho-K lens for 24 h, despite the small central reshape zone due to this short duration of lens wear, the right eye (control eye) achieved a mean keratometry change of −1.13 D within an irregular shape zone of 3.84 mm^2^ (length × width, 3.53 × 1.86 mm), versus −2.16 D within a nearly-circular zone of 3.16 mm^2^ (length × width, 2.00 × 2.04 mm) in the left eye (treatment eye). Corneal flattening was more obvious in Monkey B with overnight Ortho-K lens wear for 7 days, with a mean keratometry change of −3.48 D achieved in the right eye (control eye) within a round reshape zone of 9.27 mm^2^, versus −2.45 D within a round reshape zone of 6.87 mm^2^ in the left eye (treatment eye).

In Monkey A, regressions of corneal shape were observed in both CXL treatment and control eyes. By day 8, the control eye regressed to its original shape (mean keratometry change of −0.07 D compared to baseline in the reshape zone), while the central corneal area in the CXL treatment eye remained flat (mean change of −1.01 D in the reshape zone). At day 27, a change of −1.48 D of corneal flattening still remained in the treatment eye relative to its baseline status.

In Monkey B, the control eye had completely regressed to baseline level (mean change of −0.17 D in the reshape zone) by day 6, whereas the effect of central flattening in CXL treatment eye still remained (mean change of −1.28 D in the reshape zone) till day 14 when it returned to baseline shape (mean change of 0.21 D in the reshape zone).

The mean keratometry of reshape zone and the difference between follow-up and baseline for two monkeys are presented in detail in Fig. 3. After Ortho-K wear an abrupt decrease in corneal curvature of reshape zone occurred, followed by a gradual regression in all eyes. Eight days post CXL procedure in Monkey A, the mean keratometry of the control eye returned to baseline level, while that of CXL treatment eye decreased. In Monkey B, both eyes regressed to baseline level on day 10 post procedure, with a slower pace in the CXL treatment eye than the control eye.

### Refraction

**Figure 4:**
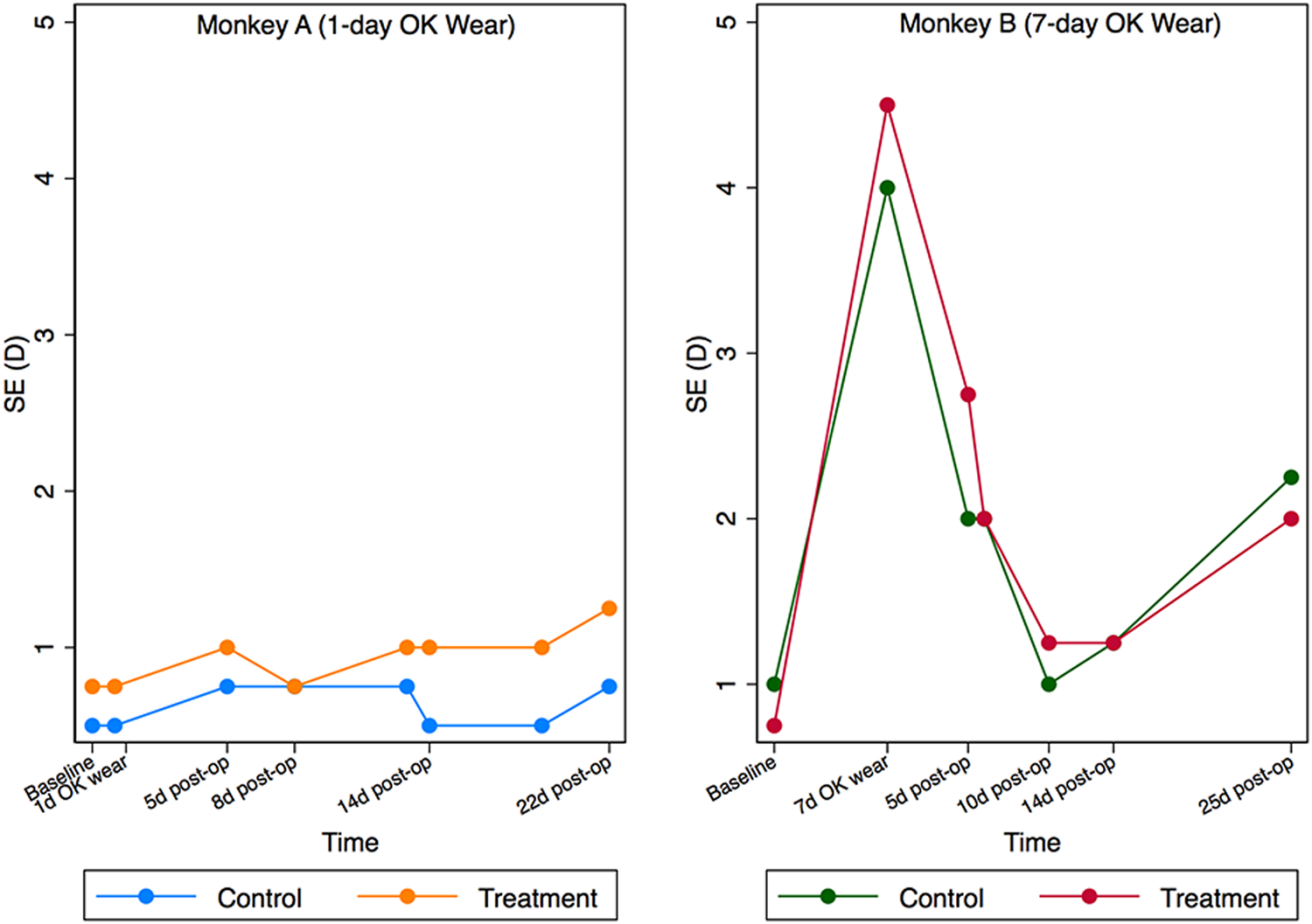
Spherical equivalent (SE) changes before and post operation. The left one portrays the SE changes (D) in monkey A (24 h lens wear), while the right one shows the SE changes (D) in monkey B (7-day lens wear). Refraction change between control and corsslinking (CXL) eye showed similar trend within the same monkey.

Figure 4 displays the retinoscopy refraction change in the two monkeys. No clinically significant refraction change was observed in Monkey A (left) while Monkey B displayed a hyperopic change of 3–3.75 D (right) as a result of corneal flattening. The refraction change was similar in the CXL treatment and control eyes during the regression phase.

## Discussion

Our study demonstrates that the current standard CXL technique may slow the regression of corneal shape molded by Ortho-K in normal corneas in rhesus monkeys, however it appears that CXL cannot stabilize the Ortho-K effect longer than one month.

The combined therapy of Ortho-K and CXL has already been applied in some previous studies, but not for myopia treatment. In clinical experiments for keratoconus patients wearing Ortho-K lenses, only temporary effects of corneal flattening were observed following CXL^10,11^. It is difficult to draw direct comparisons of Ortho-K efficacy in keratoconic and non-keratoconic eyes due to the differences in stromal structure. Our study was supported by the contralateral eye as a control, and it showed that the standard CXL procedure failed to stabilize the corneal shape molded by Ortho-K in normal cornea. While residual corneal flattening in the treated eye with 24 h of Ortho-K lens wear was observed, the associated refraction changes warrants further validation in more samples.

We hypothesize that the failure of CXL to stabilize the corneal reshape effect by Ortho-K may be attributable to several possible reasons. Firstly, although it is yet conclusive that the effect of Ortho-K is mainly attributable to the corneal epithelial re-distribution^12–14^, other studies suggested the changes of corneal posterior surface flattening^15^, stromal thinning^16^ and shorter anterior chamber depth after Ortho-K^17^. On the other hand, one study suggested that CXL might bring about structural changes in the stroma but these changes were mainly on the anterior 200 μm thick portion^18^. This might also explain the insufficient stabilization effect of CXL.

Another possible explanation for this negative result is related to the intensity of crosslinking. In our experiment, we followed the most commonly used protocol of CXL for the treatment of keratoconus^19^. However, the effect of CXL is associated with total absorbed radiant exposure, concentration distribution, and treatment time^20,21^. Though the Dresden protocol is currently the most effective way to conduct UV-A mediated CXL, issues regarding efficacy, such as pulsing versus continuous wave light, accelerated versus conventional CXL, are still in debate. Besides, other novel CXL techniques including riboflavin concentration-controlled method which is more theoretically efficient than the Dresden protocol^22^, the rose bengal-green light CXL which was proved to have a sgreater density of CXL than UV-A CXL^23^, and the novel femtosecond laser CXL to correct vision^24^, have been investigated and developed. In light of this, the intensity of CXL in our study may fall short of adequacy, but with the potential to be improved to create long-term corneal changes. However, it must be noted that methods to strengthen CXL, such as using higher energy doses, may damage the underlying structures^25,26^, hence the appropriate trade-off between efficacy and safety of crosslinking when combined with Ortho-K requires further evaluation.

The topography showed clear profile of the changes in corneal shape from re-modelling to regression, but the refraction changes were not completely in line with the topographic measurements. Although measurement showed that a flattening of −1.13 D to −2.16 D was achieved in the reshape area in Monkey A, a lack of hyperopic shift was observed. One possible explanation is that the flattening area in monkey A was relatively small. Optometrists were easily misled by the surrounding areas where no curvature change occurred. Furthermore, measurement errors of retinoscopy should be noted for the discrepancy between topography and refraction measurement.

Although the effect of CXL treatment faded over time, corneal flattening of −1.48 D remained in the CXL treated eye in Monkey A. One of the confounding factor is the potential effect on corneal flattening solely by CXL itself, since previous studies have reported mild corneal flattening and myopia reduction in keratoconus patients after CXL treatment using the current protocol^5,27,28^, though most of which was observed in steeper cornea^29^. Elling *et al*.^30^ have performed CXL alone in myopic patients with normal corneas and observed the mean keratometry changed from 42.64 D at baseline to 42.11 D at 1 month post-op. However, the energy they used was 2–3 times higher than that in our study (10–15 J/cm^2^ vs. 5.4 J/cm^2^). Therefore, we do not think the effect of corneal flattening caused by CXL alone could be excluded, but it was of much less significance in the current protocol.

Interestingly, residual corneal flattening was only observed in Monkey A. It is conceivable that different Ortho-K modalities may influence the effect of CXL. Although high oxygen transmission materials are developed and applied in Ortho-K lenses, there is evidence that ocular surface is under additional hypoxic stress during overnight Ortho-K lens wear^31^. Therefore, we speculate that long-term wear of Ortho-K lenses may lead to more severe hypoxia and corneal swelling response, resulting in the diminished effect of CXL. Moreover, biomechanical changes with increasing duration of lens wear might also be attributable to the discrepancy in CXL effect^32^. Additional data is required to further elucidate the mechanistic hypotheses for this finding.

While the current study showed that CXL failed to sustain the corneal shape molded by Ortho-K lens, it was able to retard its regression. Therefore, reducing the frequency of lens wear while prolonging the effects of Ortho-K, especially for those with low compliance, is of substantial patient benefit. However, a more suitable protocol to achieve a better outcome and less complication is needed before its application in clinical practice.

The strengths of this study include the design of contralateral eye as a control, the use of customized Ortho-K lenses and a standardized protocol for CXL, and thorough quality control of the clinical measurements. A number of limitations must also be considered. Firstly, only two rhesus monkeys were included and assigned to different Ortho-K modalities, which may have limited the generalizability of our findings. However, given the similarities of structure, shape and size in monkey and human corneas, monkeys are the most relevant animal model available to investigate the combined treatment effects of Ortho-K and CXL. We observed consistent results in both monkeys with the application of various durations of Ortho-K wear, which would therefore provide strong evidence to support our conclusion. Another possible limitation was the short duration of follow-up that may have failed to capture the long-term effect of CXL, as some studies have observed the effects of CXL treatment on keratoconus eyes years after the procedure^33^. Although the latency and duration of CXL treatment effect remained unknown, our experiment confirmed that effect of CXL on corneal shape molded by Ortho-K faded within a month, hence treatment effect was unlikely even if follow-up duration was extended further.

## Conclusions

In conclusion, standard CXL procedure failed to stabilize the corneal shape molded by short-term Ortho-K wear in rhesus monkeys. However, whether or not this combined therapy could achieve satisfactory outcome under other circumstances, including different Ortho-K modalities or CXL protocols, needs to be validated in further study. New research to develop a novel cross-linking technique for the stabilization of Ortho-K is ongoing.

## Methods

### Experimental design

This was a proof-of-concept study designed to establish the efficacy of CXL in stabilizing corneal shape following Ortho-K wear. Two male rhesus monkeys (*Macaca mulatta*) aged 2-year-old were used as subjects in these experiments. The monkeys were fitted with Ortho-K lenses bilaterally with different modalities to establish a transient or stable remolding status: Monkey A wore the Ortho-K lens continuously for 24 hours, while Monkey B wore the Ortho-K lens for 14 hours per day (6:00 PM to 8:00 AM) for 7 consecutive days to simulate habitual Ortho-K lens use in humans. This setting aims to test the efficay of CXL to stabilize the corneal shape at any time with any status. Ambient lighting was minimized and shade cloths were used on the cages in order to maximize adherence to the protocol and simulate the conditions most human users of Ortho K would experience.

Following contact lens removal, the left eye of each monkey was arbitrarily chosen to undergo CXL, while the fellow eye served as the control. Slit-lamp microscopy, corneal topography (Medmont E300 Corneal Topographer; Medmont Pty Ltd., Victoria, Australia) and retinoscopy refraction measurements (streak retinoscope; Lifecare Medical Equipments Co., Ltd., Zhejiang, China) were performed after the recovery of corneal epithelium in treated eyes and repeated at 1-week interval for a duration of 1 month. Differences between the treated and control eyes were observed.

The use of the animals in this study and protocols were approved by the Animal Ethics Committee of Sun Yat-sen University. The study was conducted in compliance with the Association for Research in Vision and Ophthalmology statement for the use of animals in ophthalmic and vision research.

### Ortho-K lenses and lens fitting

Spherical reverse-geometry gas-permeable rigid contact lenses (E&E Optics Ltd, Hong Kong), with a refractive power of −6.00 diopters (D), an overall diameter of 10.6 mm, and an optic zone diameter of 6 mm, were used in this study. The radius of lens central curvature was chosen based on the corneal curvature map captured by a corneal topographer. Additional assessment of lens fitting was conducted with fluorescein staining under slit lamp biomicroscope by an Ortho-K expert (YX). Given that monkeys are more active than human and the lenses tend to drop out easily, mobility and stabilization of the lens were the primary consideration when selecting the lenses, hence those with optimal fluorescein patterns were selected as the final fitting lenses.

### Corneal cross-linking

Standard CXL was conducted according to the Dresden Protocol^19^. The cornea underwent epithelial debridement with toothed forceps and sterile gauze, followed by 20 minutes’ soaking with 0.1% riboflavin (Avedro Inc., Waltham, MA, USA). Following this, the cornea was rinsed with a balanced salt solution prior to the application of UV-A irradiation. The cornea was exposed to UV-A light of 3 mW/cm^2^ intensity, emitted by a KXL I UV-A source (Avedro Inc., Waltham, MA, USA) for 30 minutes, receiving a total energy dose of 5.4 J/cm^2^. In addition, drops of riboflavin were added every 5 minutes during the exposure period to ensure sufficient supply to cornea. Antibiotic ointment (Tobradex^®^, Alcon, Fort Worth, USA) was administered prophylactically to the cornea directly after CXL treatment to prevent infection.

### Corneal topography

Changes in the corneal shape were monitored with the Medmont E300 Corneal Topographer (Medmont Pty Ltd., Victoria, Australia). Only pictures with a systematic comprehensive score exceeding 85 points were considered acceptable. Tangential maps and subtractive maps were used to display changes in corneal shape, while the mean keratometry of the reshape zone was provided as a quantitative measurement.

Of note, the tear films of the monkey break up easily, especially in the condition of anesthesia, thus affecting the quality of topography images. Secondly, the fixation of the monkey was not stable and hard to modulate, making the comparisons between measurements unreliable. To minimize the variability of topographic measurements mentioned above, we used saline solution and artificial tears to wet the cornea and keep the integrity of the tear films before measurements. We also tried to adjust the head position of the monkeys to capture the images only when the fixation was at/near the center. In addition, we analyzed the curvature changes in central reshape zone where the measurements were most accurate and precise.

The calculation of mean keratometry of the reshape zone was performed as follows (See Supplementary Materials). Firstly, the reshape zone of Ortho-K was identified using the tangential subtractive maps before and after Ortho-K lens wear. In subtractive maps, the reshape zone is located in the central circular zone and surrounded by the ring of mid-peripheral corneal steepening. According to a previous study^34^, it can be effectively determined using a custom step size setting of 0.1 D in Medmont Studio 6 software (Medmont Pty Ltd., Victoria, Australia). The shape of the reshape zone was portrayed and copied in each tangential map with Adobe Photoshop software. Maps with painted reshape zones were then imported into Image-Pro Plus software (IPP; produced by Media Cybernetics Corporation, USA) and the reshape zone was set as the region of interest (ROI). Using the “counting tool” in IPP, the area ratio (a_i_) of each pseudo color (i) within the ROI was estimated separately and their corresponding keratometric reading (p_i_) was verified with the color scale. The mean keratometry of ROI (P) was calculated as:

$${\rm{P}}=\mathop{\sum }\limits_{i=1}^{n}{p}_{i}\times {a}_{i}$$

### Refraction

Refraction was performed following the instillation of tropicamide drops (1%) in each eye, using a streak retinoscope. Pupillary dilation and the absence of a pupillary light reflex was considered as successful cycloplegia. The refraction examination was conducted independently by two experienced optometrists. Results with a discrepancy ≤0.5 D between the optometrists were considered valid, otherwise a third examination was required.

All measurements including lens fitting were performed 15 minutes after general anesthesia with an intra-muscular injection of pentobarbital sodium (10 mg/kg) and topical administration of proxymetacaine hydrochloride 0.5% eyedrops (Alcaine; Alcon Laboratories Inc, Fort Worth, TX).

### Description and calculations

Corneal topography profiles for baseline and each follow-up were stuck in alignment to reflect corneal shape change. The change of mean keratometry in the reshape zone was plotted with line charts and the difference in mean keratometry between follow-up examinations and baseline was plotted with bar charts. All calculations were performed using STATA Statistical Software: Release 12.0 (StataCorp LP, Colleage Station, TX).

## Supplementary information

Supplementary Material

## Data Availability

The datasets generated during and/or analysed during the current study are available from the corresponding author upon reasonable request.
