# Supplementary material for: Effects of corneal crosslinking on corneal shape stabilization after orthokeratology
Source: Sci Rep. 2020 Feb 11;10:2357. doi: 10.1038/s41598-020-59157-2 (PMC7012905; doi:10.1038/s41598-020-59157-2)
Supplement: Supplementary file 1 — Supplementary Material [file 41598_2020_59157_MOESM1_ESM.docx]

**Supplementary material: Steps to calculate mean keratometry**

**Manuscript title: Effects of corneal crosslinking on corneal shape stabilization after orthokeratology**

**Authors:** Chimei Liao^1^, Xingyan Lin^1^, Stuart Keel^2^, Jason Ha^3^, Xiao Yang^1*^, Mingguang He^1*^

* co-corresponding author

**Institutions:**

1. State Key Laboratory of Ophthalmology, Zhongshan Ophthalmic Center, Sun Yat-sen University, Guangzhou, China.
2. Center for Eye Research Australia; Ophthalmology, Department of Surgery, University of Melbourne, Melbourne, Australia.
3. Faculty of Medicine, Nursing and Health Sciences, Monash University, Clayton, Australia.

**Correspondence:**

Mingguang He

State Key Laboratory of Ophthalmology, Zhongshan Ophthalmic Center, Sun Yat-sen University, Guangzhou 510060, China

Email: [mingguang_he@yahoo.com](mailto:mingguang_he@yahoo.com)

Step 1: The reshape zone of Ortho-K was identified using the tangential subtractive maps before and after Ortho-K lens wear, using a custom step size setting of 0.1D in Medmont Studio 6 software. The reshape zone is located in the central circular zone and surrounded by the ring of mid-peripheral corneal steepening.


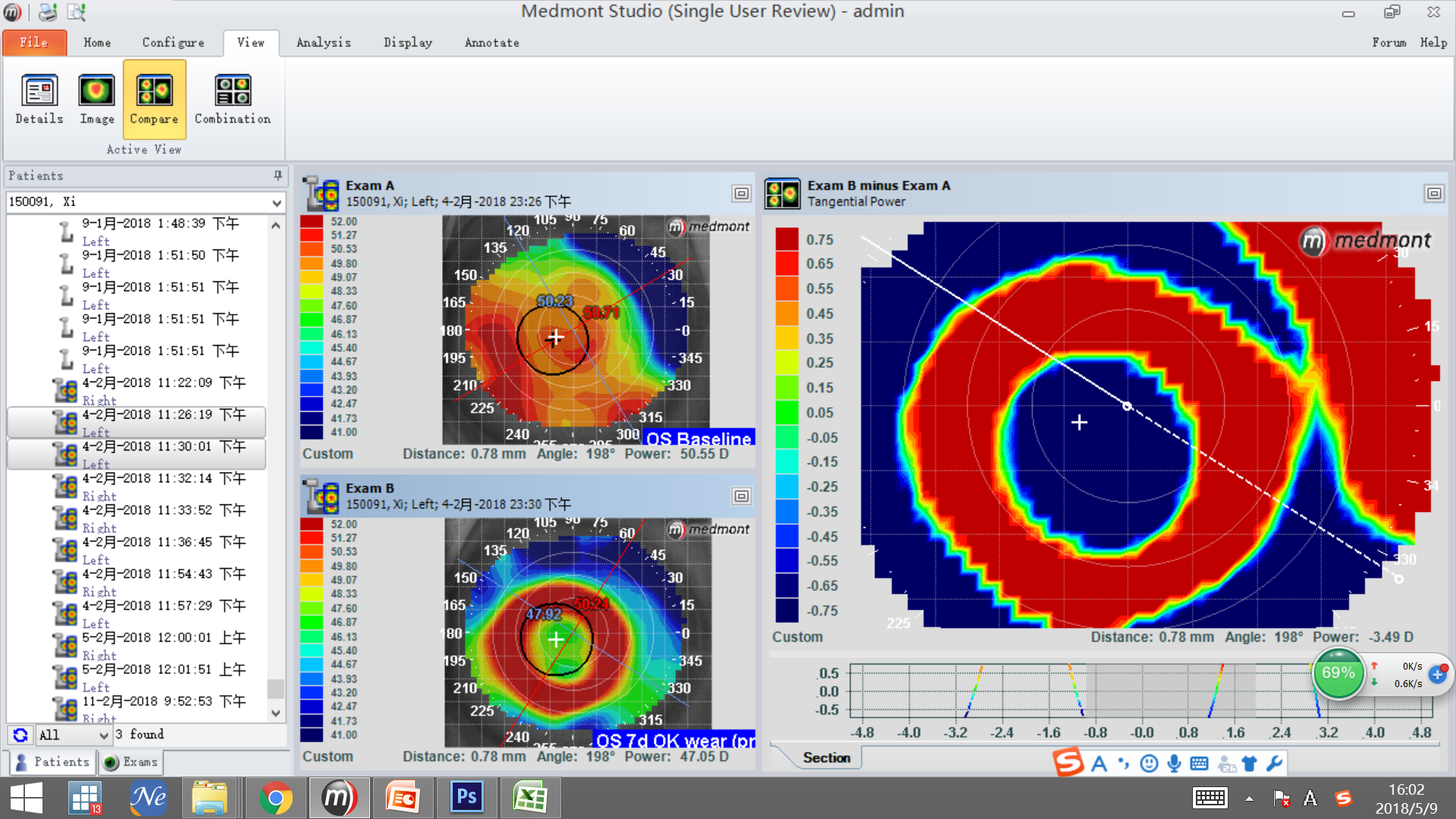


Step 2: Take a screenshot of the subtractive map and import it into Photoshop software. The green colors in subtractive maps represent keratometric change within ±0.05D. Outline the contour of the reshape zone along with the green color.


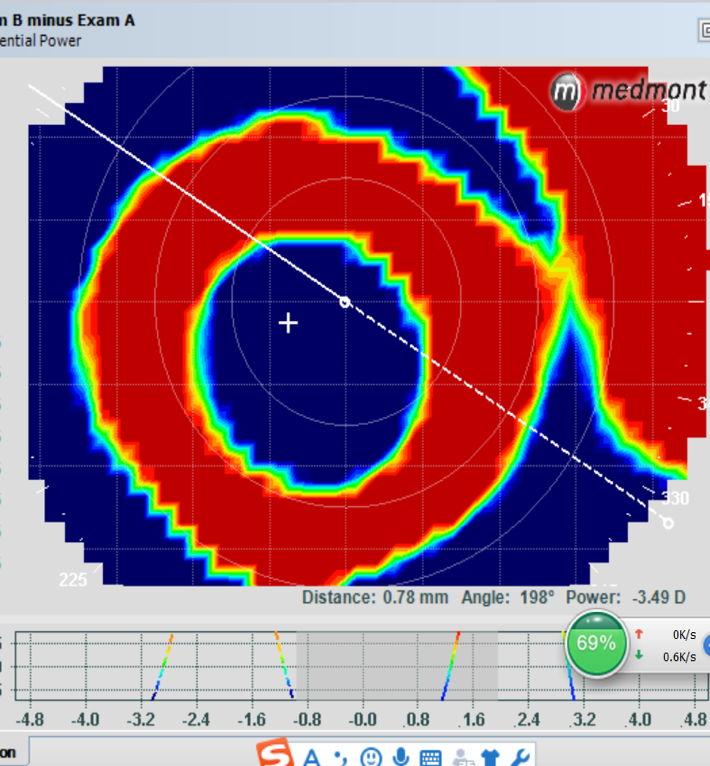


Step 3: Portray and copy the shape of reshape zone in tangential maps. Using the center of the pupil as a reference position and the grid of maps to restore the size of the zone.


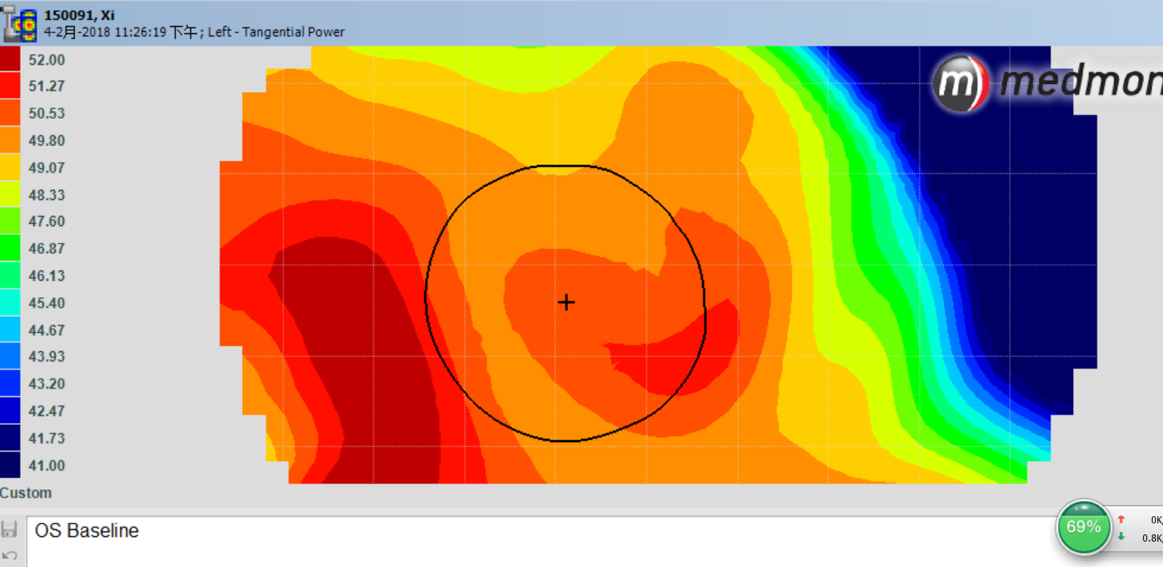


Black “+” is the pupillary center, as a reference position


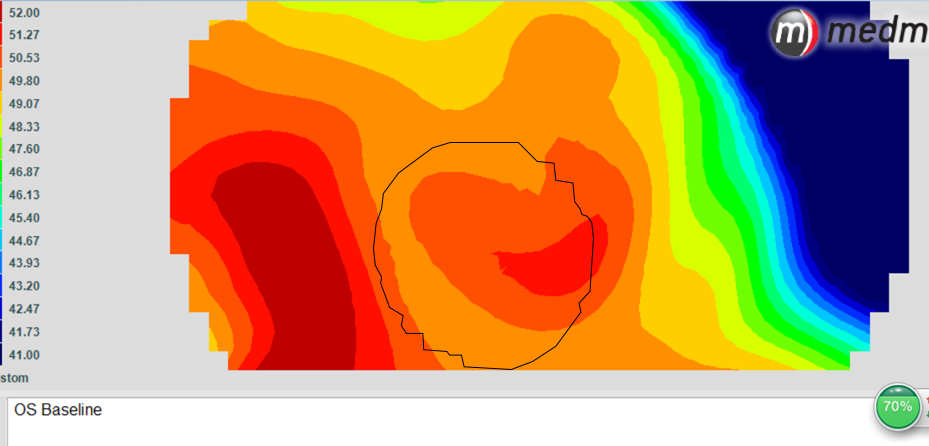


The reshape zone was portrayed and copied in this map.

Step 4: Save the maps with the reshape zone and import them into Image Pro Plus software. Using the “counting tool” in IPP, the area ratio (ai) of each pseudo color (i) within the ROI was estimated separately and their corresponding keratometric reading (pi) was verified with the color scale. The mean keratometry of ROI (P) was calculated as:

P = $\sum_{i=1}^{n} p_{i\times}a_{i}$


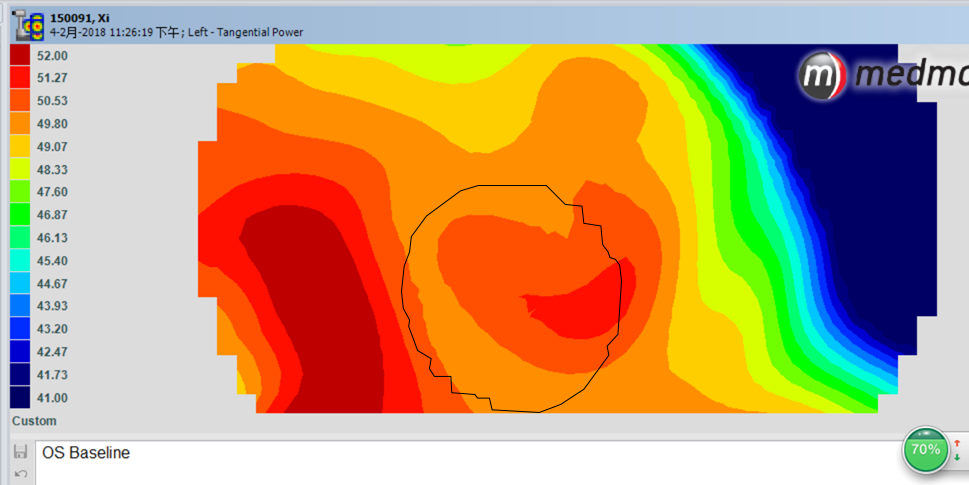


There are 3 colors existing in this ROI. Refer to the color scale and find out their corresponding keratometric readings: 51.27D, 50.53D and 49.8D. And then calculate their area and ratio.

| ID | day | EYE | UNIT | AREA (pixel) | Proportion | POWER (Area proportion * ketratometry) |
| --- | --- | --- | --- | --- | --- | --- |
| 150091 | baseline | OS | 51.27 | 4109 | 0.108243 | 5.549602 |
|  |  |  | 50.53 | 18752 | 0.493981 | 24.96084 |
|  |  |  | 49.8 | 15100 | 0.397777 | 19.80928 |
| SUM |  |  |  | 37961 | 1 | 50.31972 |

So the mean keratometry of this eye at baseline was 50.32D.
